# Audit of Sub-Therapeutic Dosing of Methadone as Opioid Substitution Therapy

**DOI:** 10.1192/bjo.2024.645

**Published:** 2024-08-01

**Authors:** Paul Wilson

**Affiliations:** Newcastle Treatment and Recovery, CNTW, Newcastle Upon Tyne, United Kingdom

## Abstract

**Aims:**

Clinical guidance indicates that methadone doses of 60–120mg are therapeutic as opioid substitution therapy (OST). Audit was completed to understand why patients open to Newcastle Treatment and Recovery (Addictions) are being prescribed doses below 60mg and to identify areas for improvement.

**Methods:**

285 patients were identified via prescription software as currently prescribed <60mg methadone. A random sample of 50 cases was obtained for audit during signing of routine prescriptions. Case sample was adjusted to ensure even distribution between keyworkers. Review then completed of prescribing card and clinical entries in the last 6 months. Standards included reason for subtherapeutic dosing and evidence of instability with use of illicit opioids, or other substances (excluding alcohol or cannabis), alongside secondary outcomes.

**Results:**

54% of cases were found to currently be undergoing a change in their dose – mostly reducing though 2 increasing and 2 preparing to switch to buprenorphine. The remaining 46% were maintained on a consistent dose of methadone below 60mg. Of these 8 were advised to change their dose but this was declined. The remaining 15 had no additional advice recorded and remained on sub-therapeutic dose. Of 50 cases 8 were unstable with regards illicit opioid use, 21 were using other substances (1 gabapentin with the remaining using cocaine). For those using illicit opioids 63% were advised of an increase but declined whilst 25% were not advised of any change in their OST. Of those using other substances 48% had no change in OST considered whilst a further 10% continued with a reduction.

**Conclusion:**

The audit found that a proportion of cases prescribed a sub-therapeutic dose were being maintained on this dose. Most concerning was the proportion of patients who were not advised to increase despite use of illicit opioids but also the proportion who were not following advice from their keyworker. Additional concerns highlighted uncertainty in practice around the role of OST in those who remain using other substances, in particular cocaine. Department of Health guidance recommends that doses in these cases should be optimised which would mean at least targeting therapeutic range. Recommendations made included to develop further training to ensure consistency of practice as well as requiring that all patients on sub-therapeutic doses of methadone should be booked for strategic care plan reviews at a minimum of 6 monthly.

